# The role of dystrophin isoforms and interactors in the brain

**DOI:** 10.1093/brain/awae384

**Published:** 2024-12-02

**Authors:** Konstantina Tetorou, Artadokht Aghaeipour, Simran Singh, Jennifer E Morgan, Francesco Muntoni

**Affiliations:** Developmental Neurosciences Department, Dubowitz Neuromuscular Centre, University College London, Great Ormond Street Institute of Child Health, London WC1N 1EH, UK; Developmental Neurosciences Department, National Institute for Health Research Great Ormond Street Hospital Biomedical Research Centre, London WC1N 1EH, UK; Developmental Neurosciences Department, Dubowitz Neuromuscular Centre, University College London, Great Ormond Street Institute of Child Health, London WC1N 1EH, UK; Developmental Neurosciences Department, National Institute for Health Research Great Ormond Street Hospital Biomedical Research Centre, London WC1N 1EH, UK; Developmental Neurosciences Department, Dubowitz Neuromuscular Centre, University College London, Great Ormond Street Institute of Child Health, London WC1N 1EH, UK; Developmental Neurosciences Department, National Institute for Health Research Great Ormond Street Hospital Biomedical Research Centre, London WC1N 1EH, UK; Developmental Neurosciences Department, Dubowitz Neuromuscular Centre, University College London, Great Ormond Street Institute of Child Health, London WC1N 1EH, UK; Developmental Neurosciences Department, National Institute for Health Research Great Ormond Street Hospital Biomedical Research Centre, London WC1N 1EH, UK; Developmental Neurosciences Department, Dubowitz Neuromuscular Centre, University College London, Great Ormond Street Institute of Child Health, London WC1N 1EH, UK; Developmental Neurosciences Department, National Institute for Health Research Great Ormond Street Hospital Biomedical Research Centre, London WC1N 1EH, UK

**Keywords:** dystrophin, brain, protein interactions, duchenne muscular dystrophy, brain comorbidities

## Abstract

Dystrophin is a protein crucial for maintaining the structural integrity of skeletal muscle. So far, attention has been focused on the role of dystrophin in muscle, in view of the devastating progression of weakness and early death that characterizes Duchenne muscular dystrophy. However, in the last few years, the role of shorter dystrophin isoforms, including development and adult expression-specific mechanisms, has been a greater focus.

Within the cerebral landscape, various cell types, such as glia, oligodendrocytes and Purkinje, cerebellar granule and vascular-associated cells express a spectrum of dystrophin isoforms, including Dp427, Dp140, Dp71 and Dp40. The interaction of these isoforms with a multitude of proteins suggests their involvement in neurotransmission, influencing several circuit functions.

This review presents the intricate interactions among dystrophin isoforms and diverse protein complexes across different cell types and brain regions, as well as the associated clinical complications. We focus on studies investigating protein interactions with dystrophin in the past 30 years at a biochemical level. In essence, the brain's dystrophin landscape is a thrilling exploration of diversity, challenging preconceptions and opening new avenues for understanding CNS physiology. It also holds potential therapeutic implications for neurological complications involving brain dystrophin deficiency. By revealing the molecular complexities related to dystrophin, this review paves the way for future investigations and therapeutic interventions for this CNS aspect of Duchenne muscular dystrophy.

## Teaser

Tetorou *et al.* discuss the role of dystrophin in the brain, beyond its function in maintaining the structural integrity of skeletal muscle. They explore how dystrophin isoforms interact with other proteins, and consider how understanding these interactions could help in treating neurological complications in Duchenne muscular dystrophy.

## The *DMD* gene and its protein products

**Figure 1:**
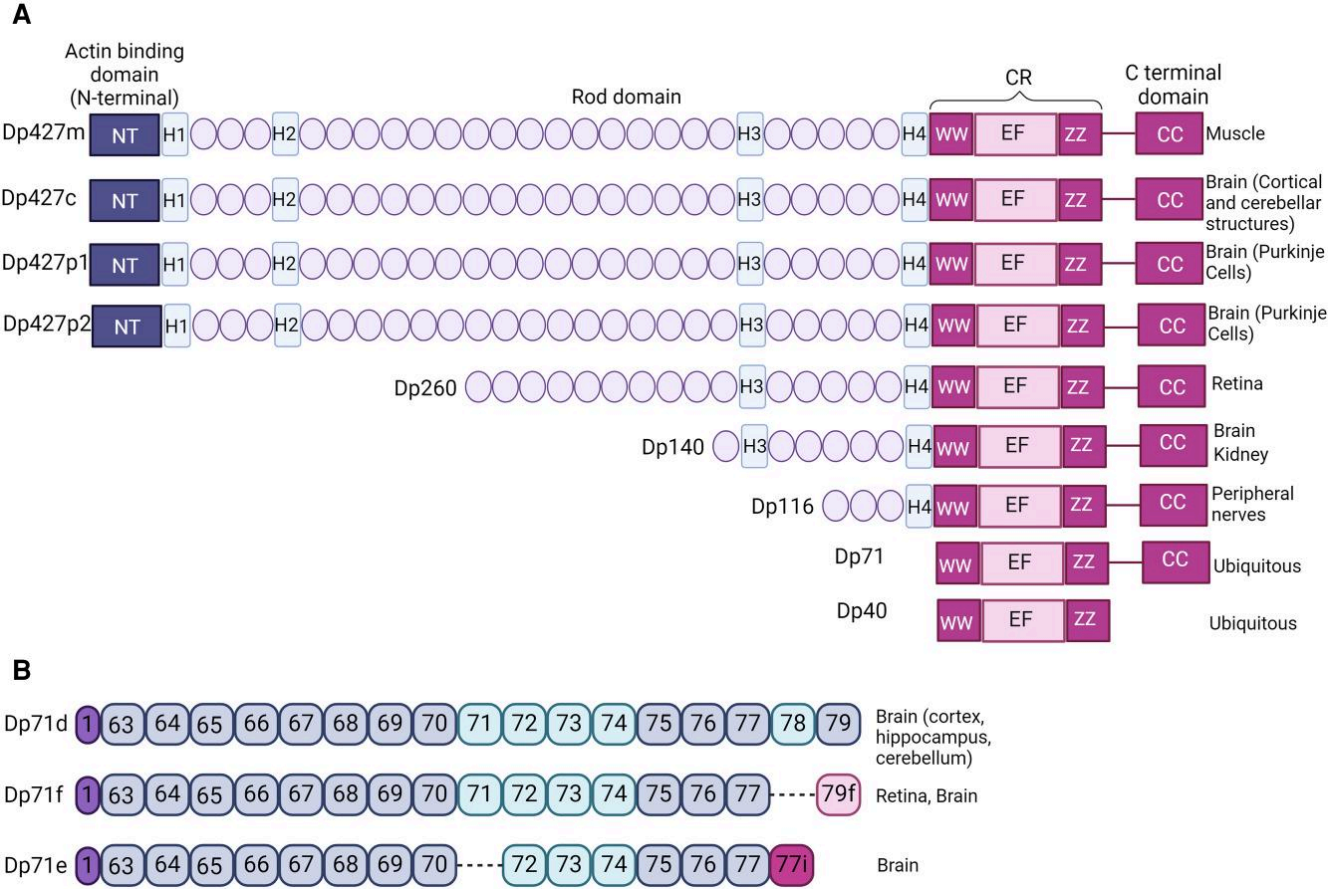
**Modular organization of dystrophin and its isoforms**. According to the UniProt database (UniProt ID: P11532) all dystrophin isoforms share a common modular structure comprising the cysteine-rich (CR) domain, while some of the isoforms also share the N-terminus (NT) with actin-binding sites from amino acid 1–240, a long central rod domain composed of β-spectrin-like repeats from amino acid 339–1463 and 1468–3040 and proline-rich hinge regions (H1–H4), predicted to form triple-helical coiled-coils, and additional domains are found in the C terminal region, including the WW domain from amino acid 3055–3088 (WW), the ZZ domain from amino acid 3308–3364 (EF/ZZ) within the CR region and the coiled-coil domain (CC) in the C-terminus domain from amino acid 3260–3685. (**A**) The C-terminal region interacts with specific components of the dystrophin-associated protein complex through these protein domains such as β-dystroglycan, while actin binds to the rod domain. Notably, the Dp427 isoform and shorter variants differ in the length of their rod domain and the high affinity actin binding domains located in the NT. Within the shorter isoforms, Dp260 is the only isoform that retains the low affinity actin binding mediated by the rod domain.^5^ (**B**) Schemes showing Dp71d, Dp71f and Dp71e, which are different due to the skipping of exon 78 in Dp71f, resulting in the lack of this exon, leading to the change of the reading frame and production of exon79f. Dp71e on the other hand lacks Dp78, 79 and 79f and retains the intron 77.^6^ This figure was generated using NCBI's Sequence Viewer and Protein tools.^7^ Figure created with Biorender.com.

The *DMD* gene is located at chromosome Xp21.2 and is responsible for Duchenne muscular dystrophy (DMD), the most common muscular dystrophinopathy of childhood. The gene consists of 79 exons and seven promoters, each associated with unique first exons.^1-3^ Among the various isoforms of dystrophin, the full-length isoform (427 kDa dystrophin protein; Dp427) can be transcribed through three different tissue-specific promoters, namely Purkinje cells, muscle and cortical structures in the brain (Fig. 1A).^4^

In addition to Dp427, shorter dystrophin isoforms (Dp260, Dp140, Dp116, Dp71) are produced via internal independent promoters located downstream of intron 29; each of these promoters uses a specific first exon, which is spliced into exons 30, 45, 56 and 63 to generate the shorter isoforms.^2,8^ These isoforms are subjected to alternative splicing, exhibiting multiple variants and tissue-specific expression patterns.^2^ Dp71 has three gene products (Dp71f, Dp71d and Dp71e) through alternative splicing events involving exons 71–74, 78 and intron 77, generating three Dp71 isoforms with specific C-terminals as a result of a frameshift induced by splicing of exon 78 and/or intron 77. Dp71d retains the last 13 amino acids encoded by exon 78, while in Dp71f exon 78 is spliced out, and the Dp71e group contains the last 34 bp of intron 77 (Fig. 1B).^9-11^ The same promoter^12^ also codes for Dp40, which is the shortest isoform of dystrophin, sharing the same 5′-untranslated region (UTR) and the sequence for the first seven amino acids with Dp71, but the 3′-UTR is derived from within the intron 70 sequence.^13,14^ It must be noted that all of the dystrophin isoforms, apart from Dp40, share a region that codes for a unique C-terminal sequence that is subject to alternative splicing (Fig. 1A).^15^

The *DMD* gene is prone to *de novo* mutations, resulting in a group of neuromuscular disorders collectively known as dystrophinopathies. Depending on the location of the mutation along the *DMD* gene, different subsets of dystrophin isoforms are affected. All mutations affect the Dp427 isoforms expressed in adult striated muscles, providing the basis for the primary involvement of skeletal and/or cardiac muscles observed in these patients. The *DMD* gene has two mutational hotspots, one located at the 5′ end and the second in the central region of the gene, which give rise to deletions (∼65% of DMD patients) or duplications (∼10%). Smaller mutations (splice site and nonsense mutations) are more evenly spread across the entire gene.^16^ Mutations that result in the absence or very limited production of dystrophin lead to DMD (typically out-of-frame deletions or duplications), while those that yield a partially functional Dp427 protein, often an internally deleted form, cause the allelic Becker muscular dystrophy (BMD) variant (typically in-frame deletions or duplications that maintain the reading frame).^17^ Patients with BMD experience relatively milder symptoms, present at a later age and have slower progression compared to individuals with DMD.^18^ A proportion of DMD patients have intellectual disability and multiple neurobehavioural co-morbidities, depending on what dystrophin isoform(s) they are lacking,^19^ which will be discussed in more detail in the subsequent sections. *DMD* mutations involving shorter Dp isoforms and associated with a BMD phenotype also result in cognitive/behavioural abnormalities.^20^ Most of our current knowledge about the functions of dystrophin isoforms in the brain comes from studies of DMD patients or animal models of DMD, where the underlying genetic mutation disrupts the expression of one or more dystrophin isoforms. This review will, therefore, introduce DMD and its animal models and present in detail the current knowledge related to the various proteins that interact with dystrophin in the brain.

## Dystrophin and muscle pathology in Duchenne muscular dystrophy

DMD incidence is 1 in 3500–5000 male births.^21^ DMD's skeletal muscle-related symptoms typically manifest before the age of 3 years, with a gradual failure of skeletal muscle repair observed in patients in the first 10 years of life, resulting in the progressive loss of muscle fibres and their replacement by fibro-fatty connective and adipose tissue.^22-24^ The progressive weakness leads to loss of ambulation and eventually premature death due to respiratory and/or cardiac failure.^25-27^

**Figure 2:**
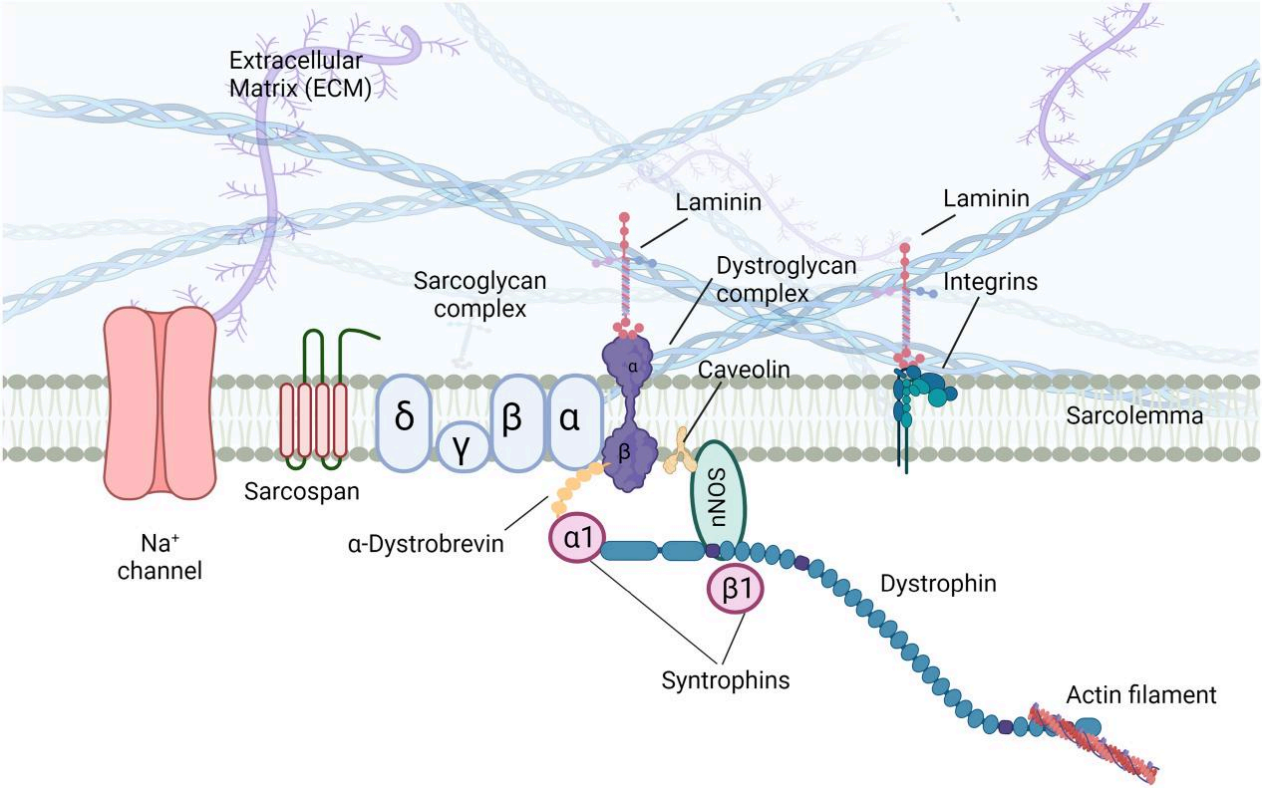
**The dystrophin complex in muscle: Dp427m, the isoform found in skeletal, cardiac and striated muscle, establishes its connections with the actin cytoskeleton through the N-terminus domain, while its C-terminus domain interacts with multiple proteins within the dystrophin-associated protein complex (DAPC)**. The figure depicts the principal structural and signalling components of the muscular complexes. Notably, α-dystroglycan binds to laminin in the muscle extracellular matrix, and the DAPC stabilizes Na+ channels in muscles. Furthermore, the DAPC in skeletal muscle encompasses the dystroglycan subcomplex, sarcoglycan-sarcospan subcomplex and cytoplasmic subcomplex, featuring syntrophins and α-dystrobrevin. The sarcoglycan-sarcospan subcomplex consists of sarcoglycans and sarcospan.^31^ Figure created with Biorender.com.

DMD is caused by mutations in the *DMD* gene that disrupt the production of functional Dp427m protein in muscles,^28,29^ resulting in an increased vulnerability to the forces generated during sarcomere contraction.^30^ In striated muscle, Dp427m is a large cytoskeleton protein that binds directly or indirectly to several proteins such as scaffolding proteins via its C-terminal domain and forms the dystrophin-associated protein complex (DAPC) (Fig. 2). The DAPC spans the sarcolemma, interacting with extracellular matrix proteins and maintaining the membrane integrity of muscle fibres, while protecting them from long-term contraction-induced damage.^32^ The following proteins form the core members of the DAPC in skeletal muscle: actin, α- and β-dystroglycan, sarcoglycans (α, β, γ and δ), syntrophins (α1 and β1), alpha-dystrobrevin (αDb) and neuronal nitric oxide synthase (nNOS).^33^

## Brain comorbidities in patients with Duchenne muscular dystrophy

A proportion of DMD patients have neurological involvement, including intellectual disability and neurobehavioural comorbidities.^34,35^ Research conducted by Bresolin *et al*.^36^ revealed that 24% of DMD patients exhibit Intelligence Quotient (IQ) levels in the normal range, while 31% have a Wechsler Full IQ below 75, suggesting mental retardation. Studies conducted by Cotton *et al*.^37^ and Battini *et al*.^38^ confirmed that the average IQ of DMD patients is one standard deviation lower than in the general population. A meta-analysis of 167 studies on DMD and BMD patients confirmed that the IQ of patients with either condition is lower than the normative value, suggesting that some BMD patients also suffer from these comorbidities.^39^

Moreover, individuals with DMD and BMD are more likely to exhibit neurodevelopmental complications, including attention-deficit/hyperactivity disorder (ADHD),^40^ autism spectrum disorder (ASD),^41^ epilepsy^42^ and anxiety disorders^43^ compared to the general population. A more recent meta-analysis of 10 studies on BMD and DMD patients concluded that patients with a *DMD* mutation that leads to the absence of Dp427, Dp140 and Dp71 isoforms have the lowest average IQ compared with patients with mutations affecting Dp140 and/or Dp427.^44^ The same meta-analysis indicated that it is plausible that participants with DMD have a lower IQ than those with BMD, although there were no statistically significant differences between BMD and DMD patients.^44^ In a detailed study of DMD patients by Ricotti and colleagues,^45^ the authors concluded that Dp427 plays a significant role in behavioural comorbidities but appears to have no effect on intellectual function. The results support the notion that mutations towards the 3′ end of *DMD*, which disrupt not only the long products but also the short brain-expressed isoforms Dp140 and/or Dp71, are associated with more profound effects on the neurocognitive phenotype.^45^ For instance, a study of 53 DMD patients showed that patients with mutations expected to abolish Dp140 expression exhibited poor performance of verbal memory and executive attention and function compared to those with intact Dp140 expression.^46^

Another study of cognitive function in 42 DMD patients lacking all the isoforms showed that all had an IQ range of 33–55, except one patient who had an IQ of 70,^20^ while in an Indonesian cohort, DMD patients lacking all isoforms had the lowest cognitive performance, with a total IQ score of 46 ± 24.8.^47^ Studies on BMD patients’ cognitive profiles are very limited; however, a meta-analysis focusing on BMD patients demonstrated that 7%–25% of the patients exhibited intellectual disability; while the patients had strong verbal working memory, many suffered from ADHD and learning disorders.^48^ Importantly, BMD patients with mutations that might alter Dp140 expression (*DMD* deletion in the 3′ end breakpoint in exon 44 or 5′ end breakpoint in exon 45) were found to perform more poorly on tests measuring verbal memory, executive functions and attention compared with the Dp140+ population.^46,49^

## Brain co-morbidities in mouse models of Duchenne muscular dystrophy

DMD occurs in various animal models, (*mdx* mice, dystrophic cats^50,51^ and dogs^52-54^) or following targeted gene inactivation, e.g. in pigs.^55,56^ The different *DMD* mutations in the dystrophic *mdx* mouse models are listed in Table 1. The findings on human brain comorbidities are supported by studies investigating the different DMD mouse models, each exhibiting varying degrees of CNS involvement. Studies focusing on *mdx23* mice lacking only the long isoform (Table 1) have reported general learning impairment, anxiety and impaired long-term memory.^57-59^ Studies on *mdx52* mice lacking both Dp427 and Dp140 indicated a higher anxiety response and learning difficulties compared with *mdx23* mice in behavioural studies^60^ as well as presenting reduced glutamatergic release in their basolateral amygdala (BLA).^60^ Research on dystrophic *mdx23* mice showed that Dp427 deficiency reduces α2-gamma-aminobutyric acid A receptor (GABAAR) expression in key brain regions such as the amygdala, cerebral cortex, hippocampus and cerebellum.^61,62^ This disruption affects GABAergic inhibitory currents, especially in the BLA, leading to abnormal fear and anxiety behaviours. In the hippocampus, altered GABAergic inhibition enhances NMDA receptor-dependent synaptic plasticity, impacting memory consolidation and motor and cognitive functions.^62-66^

**Table 1:** Murine models of Duchenne muscular dystrophy

Mouse model	Mutation	Isoform(s) affected
*Dup2*	Exon 2	Lacking Dp427
*Mdx5cv*	Exon 10	Lacking Dp427
*Mdx23*	Exon 23	Lacking Dp427
*Mdx2cv*	Intron 42	Lacking Dp427 and Dp260
*Mdx52*	Exon 52	Lacking Dp427, Dp260 and Dp140
*Mdx4cv*	Exon 53	Lacking Dp427, Dp260 and Dp140 and Dp116
*Mdx3cv*	Intron 65	Lacking Dp427, Dp260, Dp140, Dp116 and Dp71
*mdx-βgeo*	Intron 63	Lacking Dp427, Dp260, Dp140, Dp116 and Dp71
*DMD null*	Exon 63	Lacking Dp427, Dp260, Dp140, Dp116 and Dp71
*Dp71-null*	Exon 62	Lacking Dp427, Dp260, Dp140, Dp116 and Dp71

## Distribution of Duchenne muscular dystrophy isoforms in developing and adult mouse and human brain

While Dp427m is well characterized in both DMD models and human muscle, less information is available on the cell-specific localization and interactions of the various dystrophin isoforms in the brain.

Dp427c is mainly expressed in forebrain and cerebellar neurons and co-expressed with GABAARs in cornu ammonis (CA) pyramidal neurons^67^ and cerebellum.^68^ Dp427p1 and p2 are predominantly expressed in cerebellum, particularly Purkinje cells, which are integral to motor coordination.^4,69^ However, Dp71 is highly expressed in hippocampus and amygdala^69^ and is essential to astrocyte functioning,^70,71^ while it is also expressed outside the CNS.

The expression of dystrophin isoforms changes across human brain development, with low yet detectable levels of Dp427c and Dp427m during fetal development. A more recent study found Dp427p to be absent throughout brain development, in contrast with previous studies.^69,72^ High levels of Dp140 are observed in early fetal stages, and continued Dp140 expression is seen in middle adulthood in cerebellum and cortex.^69^

However, it must be noted that, although Dp140 is detected in the postnatal stages, its expression in the embryonic stages has not been fully characterized to date, since the predominant studies on Dp140 characterization have been carried out in adult mice, potentially overlooking its distinct function during the developmental phase.

Using unique transcriptomic data from the Allen Human Brain and BrainSpan atlases, Doorenweerd and colleagues^69^ determined that Dp140 exhibits very high expression during the early to mid-fetal stages (Weeks 8–10 post-conception), with no reference to earlier embryonic life, and this expression decreases significantly from the late fetal stages into adulthood in the human brain. Notably, Dp140 is most highly expressed in cerebellum, amongst all the brain regions in the adult human brain^69^; it is present in immature rat-derived oligodendrocytes and nuclear fractions as shown by *in vitro* experiments^73^ and has a significant role in glutamatergic neurotransmission.^60^ The lack of Dp140 in the *mdx52* mouse model^74^ has been linked to abnormal social behaviours and reduced glutamatergic transmission in medial prefrontal cortex–BLA projections, mirroring the ASD-like symptoms observed in DMD children who lack this isoform.^60^

Fujimoto and colleagues^75^ detected Dp71 within the hippocampal dentate gyrus of mice. They found it to be particularly abundant in the inhibitory post-synaptic compartment of granule neurons as revealed by immunohistochemistry, indicating that it is relevant to the perisomatic and dendritic post-synaptic functions. Dp71 is widely expressed during development and adulthood—its expression increasing as the brain matures.^69^ Dp71 is also detected in perivascular and forebrain astrocytes as well as cerebellar Bergmann glial (BG) cells and at the nuclear envelope.^71,76-78^ In contrast to Dp427, Dp71 is less represented in the CA, as shown by immunofluorescence detection, proposing a cell-type dependent manner of *DMD* gene expression.^75^ As explained earlier, Dp71 has several isoforms due to multiple alternative splicing events. Among these, Dp71d and Dp71f represent two subgroups in which exon 78 is present (Dp71d) or absent (Dp71f), with different cellular and subcellular distributions.^6,79^ In adult mice, Dp71f is mainly localized in the inner limiting membrane and cytoplasm of Müller glial cells in the retina, while the Dp71d isoform is localized in the nucleus, particularly the nuclear matrix, in different regions of the brain.^80^ It must be added that Dp71f is detectable in brain regions such as hippocampus, cortex and cerebellum, where it might have a role in maintaining cellular structures. Dp71d is primarily localized in hippocampus, cortex and cerebellum. The Dp71 sub-isoforms have different expression patterns during development, with Dp71f more prominently expressed during early embryonic development. As development progresses, Dp71f expression decreases while the Dp71d isoform becomes more prominent, coinciding with brain maturation.^79^

Information on Dp40 is very limited in the literature due to the difficulty distinguishing between Dp71 and Dp40, as they share a first exon. However, Dp71 + Dp40 is found to have consistent expression in both developing and adult human brain.^69^ Dp40 is localized at synaptic vesicles in the mouse brain, interacting with many proteins involved in synapses.^81^ Additionally, Dp40 has been found to localize in the subplasmalemmal regions of dendrites and the nucleus in cultured hippocampal neurons.^82^ A recent immunohistochemical study by Fujimoto *et al*.^83^ examined the expression of dystrophin isoforms in mice during brain development and found that Dp42 and Dp71 are mainly but not exclusively expressed in adulthood, while Dp140 is primarily expressed in developing mice, indicating a potential role of Dp140 in development.

## Dystrophin–protein complexes in the brain

A detailed summary of the current literature on dystrophin-protein interactions is presented in Fig. 4.

### Dp427

Several studies of adult rodent brains have reported that the Dp427 isoform associates with synaptic proteins, particularly post-synaptic proteins, indicating that Dp427 is likely localized at post-synaptic termini.

SynArfGEf coded by *IQSEC3* interacts with the Dp427 isoform as demonstrated in two-hybrid and pull-down assays on C57Bl/6N adult mouse brain.^84^ SynArfGEF mRNA is widely expressed in the rat brain as shown by *in situ* hybridization and localized in dendrites and cell bodies, proposing a post-synaptic role in the brain.^85^

SynArfGEF was initially identified as a potential synaptic guanine nucleotide exchange factor (GEF) for the ADP ribosylation factor (Arf) family of small GTPases by screening for associated mRNA species with the post-synaptic density fraction.^85^ The Arf family consists of six structurally related members (Arf1–6) crucial for membrane trafficking and cytoskeletal re-arrangements.^86^ Arf6 is the most divergent of these members, localized at the plasma membrane and endosomes and involved in the regulation of plasma membrane recycling, the peripheral actin cytoskeleton, formation and maintenance of dendritic spines, axon and dendrite branching, exocytosis and endocytosis of synaptic vesicles and receptor internalization in neurons.^87-92^

Through immunohistochemical analyses, Fukaya *et al*.^84^ showed that synArfGEF is present as puncta in somata and dendrites, closely associated with inhibitory synapses. Immunoelectron microscopy further indicated its preferential localization at post-synaptic specializations of symmetric synapses. Using yeast two-hybrid and pull-down assays, synArfGEF was found to bind utrophin/Dp427 and S-SCAM, which is also known as membrane-associated guanylate kinase inverted-2 (MAGI-2), at inhibitory synapses via a PY motif and PDZ-binding motif in the C-terminal region, respectively. S-SCAM/MAGI-2 has a probable role in the molecular organization of post-synaptic densities and synaptic transmissions. Additionally, mutations in S-SCAM are found in patients with neurological disorders such as childhood-onset schizophrenia.^93^

Immunostaining of cultured hippocampal neurons and cerebellar cortex verified the co-localization of synArfGEF with Dp427 and S-SCAM.^84^ While Dp427 and S-SCAM were immunoprecipitated from brain lysates by anti-synArfGEF immunoglobulin G, Dp427 or utrophin could not be detected in co-immunoprecipitation assays.^84^ This suggests that synArfGEF most likely binds to S-SCAM and Dp427, but not utrophin, as its physiological partners at inhibitory synapses. S-SCAM interacts directly with two inhibitory postsynaptic components, β-dystroglycan and neuroligin-2 (NL2), suggesting that synArfGEF's interaction with Dp427 and S-SCAM allows it to activate Arf6 near the dystroglycan complex (DGC) and NL2 at inhibitory postsynaptic specializations.^94^ NL2 is the only member of the neuroligin protein family that localizes exclusively at inhibitory synapses and is important in developing and establishing inhibitory synapse functions and maintaining these synapses in adulthood. A mutation in the gene encoding NL2 has been linked to ASD.^95^

**Figure 3:**
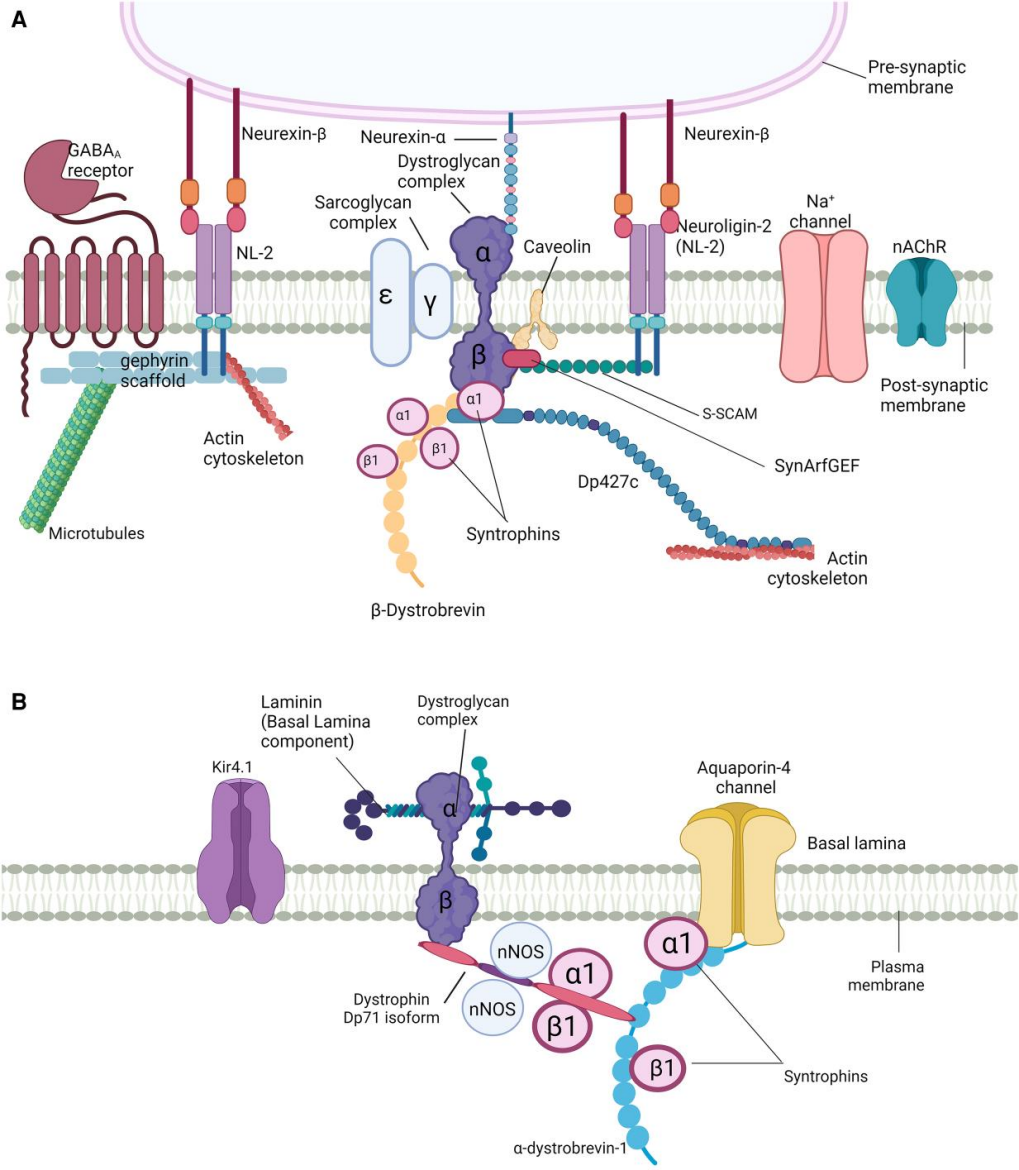
**Suggested models illustrating the dystrophin-associated protein complex (DAPC) in neurons and astrocytes.** The DAPC, specifically syntrophin and dystrophin (Dp427c), is involved in stabilizing and regulating the voltage-gated Na+ channels in both muscles and neurons. (**A**) Dystrophin and constituents of the DAPC participate in multiprotein complexes that anchor receptors to specialized membrane sites. This figure highlights the complex interplay of proteins and scaffolding molecules that ensure functional synapses and structural integrity, such as Neuronal acetylcholine receptor (NaChR). For instance, the GABAA receptor, which is involved in inhibitory neurotransmission, is anchored to the cytoskeleton via the gephyrin scaffold. While neuroligins (NL-2) and neurexins act as synaptic cell-adhesion molecules and ensure proper synaptic transmission. Dystroglycans are often co-localized with Dp427c in CNS, bridging NRX to dystrophin, while S-SCAM links the NL2 to the DGC. (**B**) DAPC complexes consisting of Dp71, dystroglycan and the syntrophins are found at the specialized end-feet processes of perivascular astrocytes, where they cluster with AQP4 and Kir4.1 channels. Dp71 has been co-immunoprecipitated with AQP4 and Kir4.1 in multiple studies.^96^ It is hypothesized that laminin potentially enhances the Kir4.1 clustering. Figure created with Biorender.

The same study showed that *mdx23* mice lacking Dp427 had reduced clustering of GABAARs containing the α2 subunit, while gephyrin clustering in the cerebellar cortex was unaffected.^84^ This points towards a Dp427-dependent and gephyrin-independent mechanism for clustering certain GABAAR subtypes (Fig. 3A).^58^ Gephyrin is a post-scaffolding molecule that plays a role in organizing the inhibitory post-synaptic density and is essential for GABAAR function.^97^ The study by Fukaya *et al*.^84^ also highlighted the potential role of synArfGEF in the Dp427-dependent clustering of GABAARs through Arf6-dependent actin cytoskeleton remodelling, thus linking Arf6 signalling pathways to inhibitory synapses and proposing its influence on dynamic processes affecting the synaptic localization of inhibitory GABAA and glycine receptors.

**Figure 4:**
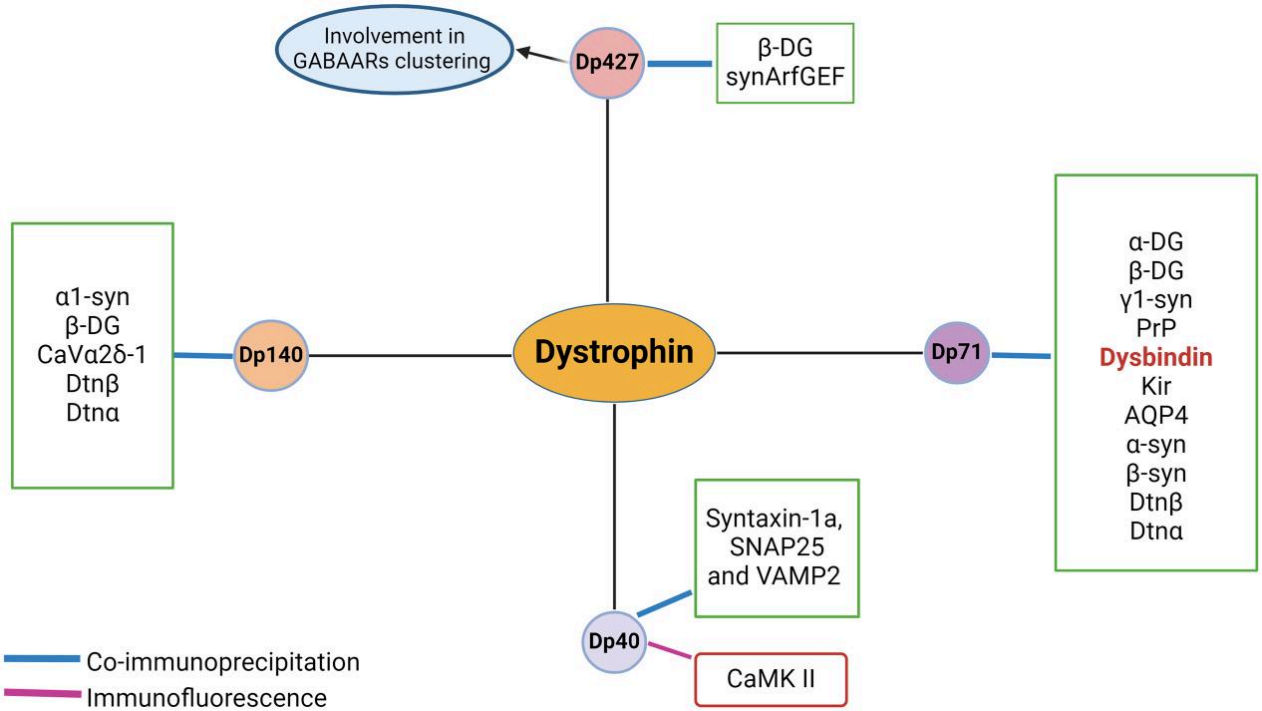
**Different dystrophin isoform–protein interactions.** Dp427 interacts with SynArfGEF (IQSEC3), which is crucial for membrane trafficking and cytoskeletal rearrangements at inhibitory synapses. The clustering of GABAARs is also dependent on Dp427, highlighting its role in synaptic stability and function. Furthermore, β-dystroglycan interacts with Dp427 at presynaptic sites, indicating a broader involvement in synaptic functions. Dp140 forms a complex with α1-syntrophin and the CaV2.1 channel, influencing neurotransmitter release at synapses and contributing to glutamatergic transmission and synaptic plasticity. Dp140's interaction with the CaV2.1 channel complex includes the CaVβ4e subunit, which regulates CaV2.1 channel activity. Additionally, Dp140 interacts with CaVα2δ-1, a crucial component in presynaptic membrane organization. Dp140 also co-immunoprecipitates with β-dystroglycan, which is a common interactor among dystrophin isoforms. Dp71 interacts with several important proteins involved in synaptic function and neurodevelopment. It binds with α-dystrobrevin and β-dystrobrevin, where β-dystrobrevin forms complexes with Dp71 in neuronal cells, and α-dystrobrevin associates with Dp71 in glial cells. Dp71 also co-localizes with α-dystroglycan in both neuronal and non-neuronal cells, forming complexes with β-dystroglycan at inhibitory postsynapses. Additionally, Dp71 interacts with various syntrophins (α1, β1, β2, γ1, γ2), which act as adaptor proteins linking Dp71 to signalling molecules. Dp71 also associates with DAMAGE protein, involved in development and cell cycle regulation, as well as Kir4.1 and Kir2.1 and AQP4, which support potassium buffering and water/ion balance. Dysbindin, implicated in schizophrenia, and the PrP, which is involved in neurodegenerative diseases, also interact with Dp71, emphasizing its role in maintaining synaptic structure and function. Dp40 interacts with Syntaxin-1a, SNAP25 and VAMP2, key SNARE proteins involved in vesicle docking, fusion and exocytosis at presynaptic sites. Dp40's role in synaptic vesicle fusion is highlighted by its interactions with these SNARE proteins. Additionally, Dp40 interacts with CaMKII, a synaptic signalling protein essential for memory formation and synaptic potentiation, indicating its involvement in Ca^2^⁺-triggered processes. α-DG = α-dystroglycan; β-DG = β-dystroglycan; α-syn = α-syntrophin; β-syn = β-syntrophin; γ1-syn = γ1-syntrophin; AQP4 = aquaporin-4; CAMK II = calcium/calmodulin-dependent protein kinase II; CaVα2δ-1 = voltage-gated calcium channel alpha2 delta subunit 1; Dtnα = α-dystrobrevin; Dtnβ = β-dystrobrevin; Kir = potassium channels; PrP = major prion protein; SNAP25 = synaptosomal associated protein 25; VAMP2 = vesicle-associated membrane protein 2. Note dysbindin highlighted in red, as there are conflicting co-immunoprecipitation results. All proteins described except CAMK II have been identified in immunoprecipitation studies. Figure created with Biorender.

A study on *mdx23* mice^98^ indicated that Dp427 is not required for the post-synaptic clustering of NL2, GABAARs and gephyrin. GABAARs are stabilized through direct interaction with a submembranous lattice of gephyrin. Similarly, in the hippocampal CA1 region, the absence of Dp427 did not eliminate the synaptic clustering of NL2 itself. However, a complex pattern of changes in the distribution of pre- and post-synaptic proteins in inhibitory synapses of *DMD-null* mice was observed, likely indicating a rearrangement of the GABAergic synaptic network. These results differed from earlier studies that showed selective deficiencies in the synaptic clustering of GABAARs, but not gephyrin, in cerebellum and amygdala of *mdx*23 mice.^61,62^ The selective loss of GABAAR, but not gephyrin, clusters is surprising, especially given that the deletion of GABAARs from Purkinje cells results in a significant defect in the clustering of gephyrin,^99,100^ without affecting dystrophin and dystroglycan.^100^ It is important to emphasize that Dp427 is crucial for normal GABAergic function in Purkinje cells, CA1 pyramidal cells and amygdala neurons.^57,62,101,102^ Along with data reported by Knuesel *et al*.,^61^ this may be explained by the fact that Dp427 contributes to stabilizing post-synaptic GABAARs by regulating the trafficking of peri/extrasynaptic receptors^103^ and that loss of the protein causes subtle effects not readily detected by immunofluorescence. At the same time, GABAARs present heterogeneity, which could be affected by the loss of Dp427. Furthermore, western blot analysis and immunofluorescence confocal imaging of *mdx23* tissue sections unveiled intricate and varied changes in the expression levels and/or clustered distribution of various synaptic and extrasynaptic GABAAR subunits across hippocampus, cerebellum, cortex and spinal cord. Consequently, the loss of Dp427 may not only impact the stability of synaptic GABAARs at specific locations, such as cerebellum, hippocampus and amygdala,^61,62,104^ but also exert an influence on the subunit composition of GABAAR subtypes at both synaptic and extrasynaptic locations.^58^

Additionally, Dp427 interacts with dystroglycan, which comprises two subunits, extracellular α-dystroglycan and transmembrane β-dystroglycan, derived from a precursor polypeptide by post-translational modifications, as shown by a cloning and sequence analysis study.^105^ α-dystroglycan and the Dp427 isoform are widely co-localized in brain, as demonstrated by immunolabelling. They are expressed in neuronal and non-neuronal cells, with predominant expression in pyramidal and Purkinje cells.^106^ Furthermore, β-dystroglycan, a key protein with versatile function^107^ that participates in cell signalling during cell polarity,^107,108^ interacts with Dp427,^106^ and it is noteworthy that this interaction occurs presynaptically, while Dp427 is mainly post-synaptic and located in inhibitory synaptic domains.^109^ This observation could be due to the presence of a subset of Dp427 proteins that is not post-synaptic but presumably interacts with different cell types, including blood vessels.

The Dp427 isoform of dystrophin plays a critical role in the brain, particularly in the context of synaptic function and organization. Its interaction with key synaptic proteins such as dystroglycan, S-SCAM and synArfGEF highlights its involvement in both excitatory and inhibitory synaptic transmission. The distinct localization of Dp427 at post-synaptic sites, coupled with its potential presynaptic interactions, suggests a multifaceted role in maintaining synaptic stability and signalling. The timing of Dp427 expression and its localization in specific neuronal populations, such as Purkinje cells, underscores its importance in neurodevelopment and synaptic plasticity.

Future research should focus on elucidating the precise molecular mechanisms by which Dp427 interacts with specific proteins involved in brain comorbidities, such as ASD, present in DMD patients. It is also important to understand the mechanism of Dp427’s contribution to synaptic clustering and the regulation of neurotransmitter receptors in different cellular populations, particularly GABAARs. Additionally, exploring the implications of Dp427 dysfunction in neurological disorders could provide valuable insights into potential therapeutic targets. Advancing our understanding of Dp427's interactions and signalling pathways will be crucial for developing strategies to mitigate synaptic pathologies associated with its deficiency.

### Dp71

The most predominant dystrophin isoform in the brain is Dp71, expressed in neurons and glia.^76^ Nuclear, subcellular localization and functions related to cellular differentiation and proliferation, as well as other cellular processes, have been described for Dp71.^110,111^ Among the dystrophin isoforms, protein interactions are mostly described with Dp71 and are summarized below. Given the apparent abundance of sub-isoforms associated with Dp71, recent characterization studies by González-Reyes *et al*.^6^ and Aragón *et al*.^79^ suggest distinct subcellular isoform expression, thus indicating a need to explore the nuanced function of Dp71 isoforms.

#### α-dystrobrevin, β-dystrobrevin, α- and β-dystroglycan

Studying Dp71 expression exclusively is challenging using immunohistological techniques, as the primary amino acid sequence of the Dp71 isoform is identical to the longer isoforms, except for its hexapeptide at the NH_2_ terminus. Hence, Fujimoto and colleagues^112^ successfully generated a novel mouse line with a specific Dp71 tag-insertion by genetically inserting DNA fragments encoding for a haemagglutinin (HA)-tag peptide at residues 1–617 for Dp71d and residues 1–622 for Dp71f to investigate the expression and localization of Dp71 protein in the adult mouse brain, focusing on hippocampus. The study demonstrated that the Dp71 isoform was present in glia limitans and exclusively in the inhibitory post synapses of hippocampal dentate gyrus granule neurons *in vivo*, as shown by immunohistochemical analysis, whereas Dp427 localizes in the CA1 pyramidal neurons.^75^ Other studies have also demonstrated that Dp427 mRNA predominates in the CA pyramidal neurons,^67,113^ displaying different spatial expressions of Dp71 and Dp427 proteins in the hippocampus.

Moreover, synapse-associated Dp71 interacts with β-dystrobrevin (βDb), dystroglycan and Inhibitory Synaptic Factor 1 (Insyn1); in contrast, glia-associated Dp71 exhibits interactions with α-dystrobrevin (αDb) and dystroglycan.^75^ Insyn1 is preferentially found with gephyrin at inhibitory post-synaptic densities and is a potential candidate gene for epilepsy and intellectual disability.^114^

Dystrobrevins share a similar structure to the C-terminal domain of dystrophin, with α-helical coiled-coils,^115^ and, like dystrophin, interact with the sarcoglycan complex and nNOS.^116^ They play an important role in intracellular signal transduction as well as providing a membrane scaffold in muscle.^116^ αDb is expressed predominantly in skeletal muscle, heart, lung and CNS and is involved in synaptic transmission at the neuromuscular junction and in intracellular signalling.^117,118^ βDb is only found in non-muscle tissues, predominantly expressed in kidney and brain, and forms complexes with DAPC and syntrophin in liver and brain. In the brain, βDb associates with Dp71 and Dp140 in cortex, hippocampus and Purkinje cells.^119^

Differential distribution of αDb and βDb in brain leads to the formation of different complexes with dystrophin and syntrophin in glial versus neuronal cells.^120^ Dp71 interacts with αDb in glial cells and with βDb in dentate gyrus granule neurons simultaneously. βDb is a neuronal, post-synaptic density-enriched, dystrophin-binding protein. In contrast, αDb-1 is found in glia, where it is primarily associated with Dp71, although the transmembrane binding partner that links these subplasmalemmal complexes to the membrane has not been identified.^120^ These data are in line with those reported by Fujimoto and colleagues,^112^ while Grady and colleagues^121^ showed that cerebellar Purkinje cells and BG cells expressed βDb and αDb, respectively.

α- and β-dystroglycan is widely co-localized with Dp71 in brain, specifically expressed in both neuronal and non-neuronal cells with predominant expression in pyramidal and Purkinje cells.^106^ The immunolocalization patterns of dystroglycan and Dp71 are consistent with the possibility that these molecules form a complex at several loci in the brain, including neuronal cell bodies and dendrites, as well as the glial interface of the blood–brain barrier.^4^ β-dystroglycan is associated to Dp71-DAPC in the nucleoskeleton fraction, demonstrated by co-immunoprecipitation studies on primary cultures of hippocampal neuronal cells,^122^ in line with other studies indicating Dp71's nuclear localization.^77,123^

A specific interaction between β-dystroglycan and Dp71 has also been studied in rabbit brain,^124^ showing that the 15 C-terminal amino acids of β-dystroglycan organize a unique binding site for the second half of hinge 4 and the cysteine-rich domain of Dp71 (amino acids 3054–3271). This dystrophin binding site is located in a proline-rich environment of β-dystroglycan within amino acids 880–895.

Affinity assays using glutathione- (GST)-agarose and GST-β-dystroglycan-agarose beads were conducted on alkaline extracts of brain microsomes to examine whether β-dystroglycan can bind to the C-terminal of dystrophin isoforms (excluding Dp40).^124^ Sodium dodecyl-sulfate polyacrylamide gel electrophoresis (SDS-PAGE) and immunoblot analysis of the proteins bound to the affinity beads were performed using affinity-purified antibodies to the C-terminal portion of dystrophin. The GST-β-dystroglycan-agarose beads, but not the GST-agarose beads, specifically bound dystrophin from brain extract, suggesting the interaction of dystrophin with β-dystroglycan. The protein highly enriched in the GST-β-dystroglycan-agarose beads compared with the alkaline extract may correspond to Dp71.^124^

In *mdx3cv* mice, the absence of all dystrophin isoforms leads to subtle changes both in behavioural outcomes^125^ and in the levels of several proteins expressed in the brain. Reduced expression of α- and β-dystroglycan was observed in cortex, hippocampus, cerebellum and spinal cord of these mice.^125^ Previous research by Greenberg *et al*.^126^ linked the decrease in β-dystroglycan expression in *mdx3cv* mouse brain to the absence of Dp71. However, considering all dystrophin isoforms are absent in *mdx3cv* mice and α-dystroglycan and β-dystroglycan are also associated with Dp427, Dp140 and Dp71 in normal mouse brain,^125,126^ drawing isoform-specific conclusions is challenging. Therefore, the reduction in α- and β-dystroglycan expression levels may indicate a decrease within each of the three complexes.^126^ It is also important to note that there are differences in the complex between mice and humans.^127^ β-dystroglycan co-localizes with Dp71 in cultured mouse neurons and dentate granule neurons, indicating their functions at inhibitory post-synapses.^75^ Dystroglycan is necessary for the proper expression and submembranous localization of Dp71 in cultured Neuro2a cells.^112^ The mechanism of this interaction entails dystroglycan anchoring to Dp71 at inhibitory post-synapses, providing a molecular scaffold to form a functional complex. In the same study, Dp71 was detected at not all but some gephyrin-positive synapses, suggesting either that physiological signalling is necessary for Dp71 clustering at specialized compartments and/or specific cell types express Dp71 in heterogenous neuronal culture. Since hippocampal neuronal culture includes pyramidal neurons, granule neurons, interneurons and glial cells,^128^ presynaptic innervations from particular GABAergic neurons might reflect their physiological situation in different states. Therefore, it is interesting that conditional deletion of dystroglycan in pyramidal mouse neurons caused loss of cholecystokinin (CCK)-positive basket cell terminals in hippocampus and neocortex,^129^ which may indicate that Dp427- and Dp71-dystroglycan complexes may be required to form and maintain CCK-positive terminals on pyramidal neurons and dentate gyrus granule neurons in hippocampus.

### Syntrophins

Syntrophins (α1, β1, β2, γ1 and γ2) exhibit specific tissue distribution, distinct sub-cellular localization and unique expression patterns, implying their diverse functional roles.^130^ They act as adaptor proteins linking signalling proteins to dystrophin; they also function as scaffolding proteins containing many protein-protein and protein-lipid interaction domains interacting with various molecules such as aquaporin 4 (AQP4).^131^

A brief summary of syntrophin interactions with dystrophin was provided earlier. In more detail, a study by Alessi and colleagues^132^ revealed that γ1-syntrophin is localized mainly in specific subsets of neurons within the brain, including hippocampal pyramidal cells, cortical neurons and cerebellar Purkinje neurons. Dp71 was observed to interact with β1-syntrophin in a 3-month-old C57Bl/6 mouse brain, while γ1-syntrophin was not detected. These data differ from those reported by Piluso and colleagues,^133^ where γ1-syntrophin was found to associate with dystrophin and other dystrophin family proteins in yeast two-hybrid assays of the same species. The binding patterns of various syntrophin isoforms indicate three distinct types of syntrophin binding sites within the dystrophin family. These binding sites exhibit varying selectivity for different syntrophin isoforms, with some binding multiple isoforms and others showing specificity for either α/β- or γ-syntrophins. This distinct binding specificity between α/β- and γ-syntrophins suggests that these two subfamilies of syntrophins are likely to have unique biological functions.^132^

In the mouse brain, γ1- and γ2-syntrophins, as well as Db, bind to dystrophin isoforms Dp71 and Dp140.^134^ In common with dystrophin and βDb, γ1- and γ2-syntrophins are found in the cortex and hippocampal formation. Additionally, Vaillend and colleagues^125^ demonstrated that syntrophin remains unaffected in post-synaptic densities and other subcellular compartments in dystrophin null *mdx3cv* mice, implying that syntrophin might interact with molecules other than dystrophin.

### DAMAGE

Dystrobrevin-associated MAGE (DAMAGE) protein encoded by *MAGEE1*, first identified by Albrecht and colleagues,^135^ belongs to the MAGE (Melanoma Antigen Gene) family of proteins, which are typically involved in various cellular processes, including development, apoptosis and cell cycle regulation. DAMAGE interacts with the N-terminal region of αDb and Dp71 isoforms. These results were identified by using a yeast two-hybrid, co-immunoprecipitation with the dystrobrevin–syntrophin complex from αDb^-/-^ mouse brain and wild-type C57Bl/6, respectively, using a pan syntrophin antibody. DAMAGE is highly expressed in brain and present in cell bodies and dendrites of Purkinje neurons.^135^

The gene encoding DAMAGE, located on the human X chromosome, may play a role in Wieacker-Wolff syndrome,^136,137^ characterized by intellectual disability, hypotonia and distinctive facial appearance. Moreover, given that DAMAGE has been identified as part of the dystrophin complex, is found in both the CNS and peripheral nerves and is located on the X chromosome in a region containing loci linked to brain comorbidities, it becomes a potential candidate gene for other unexplained conditions.^135^

### Inwardly rectifying potassium channels and aquaporin-4

Neuronal activity triggers the release of potassium ions into the extracellular space, resulting in their accumulation. Effective removal of extracellular potassium is essential for maintaining proper neuronal signalling; otherwise, it could disrupt the potassium equilibrium potential and lead to the depolarization of neighbouring cells. This process, known as spatial potassium buffering, involves glial cells, which utilize inwardly rectifying potassium (Kir) channels to transport excess potassium ions from the active neuropil to sinks.^138,139^ Kir4.1 is a transmembrane protein that exists as a tetramer and is predominantly expressed in the CNS, particularly in glial cells such as brain astrocytes^140^ and Müller cells in the retina.^141^ Notably, Kir4.1 channels are highly concentrated in the astrocytic processes surrounding blood vessels.^142^

Leonoudakis and colleagues^143^ carried out a proteomic analysis to investigate the protein interactions of Kir channels in heart, skeletal muscle and brain of adult rat. The study revealed that the Kir2-interacting proteins included members of the dystrophin-associated complex (α1-, β1- and β2-syntrophin, Dp71 and dystrobrevin; Fig. 3B). These proteins were identified by high-performance liquid chromatography-mass spectrometry (HPLC-MS) in heart extracts and confirmed in heart, skeletal muscle and brain by immunoblotting. Specifically, the use of DAPC antibodies in the brain established two isoforms of syntrophin, two isoforms of αDb, and two Dp71 isoforms of dystrophin interacting with Kir2 channels. Therefore, Kir2 channels also associate with the DAPC in brain.^143^ As other proteins of this family, Kir2 contains modular protein–protein interaction domains, therefore, and may perform a variety of unique functions in channel trafficking and localization.^143^ At the same time, Connors *et al*.^144^ demonstrated that Kir4.1 channels associate with both β-dystroglycan and Dp71 through direct binding to α-syntrophin in cultured cortical mouse astrocytes, thus indicating that the interaction of dystrophin and Kir channels may occur in astrocytes. The Kir2 protein family encompasses various subunits, including those expressed in glial and endothelial cells. This suggests that the functional role of Dp71 in the CNS may exhibit substantial variability contingent upon the isoform to which it binds. Future work to confirm the interaction between Kir2 and Dp71 will further clarify the functional significance of this proposed interaction.

The Kir channel Kir 2.1 is responsible for maintaining the resting potential of neurons.^145^ Histochemical studies showed that Kir2.1 is expressed in astrocytes, glial cells and neurons in the rat piriform cortex^146^ and at high levels in astrocytes in the olfactory bulb of adult mice but detected only under pathological conditions (induced epileptic seizure) in hippocampal astrocytes.^147^ Kir2.1 is expressed in bovine brain endothelial cells *in vitro* and upregulated by hypoxia.^148^ There is evidence from rodent microglia cells *in vitro* that Kir2.1 is involved in their proliferation and migration.^149^ Kir2.1 and 2.2 are expressed in vascular smooth muscle cells and endothelial cells in the brain, and their interactions with membrane lipids and association with caveolin 1 and syntrophin control the haemodynamic response of the vessels.^150^ Whether Kir2.1 and Dp71 interact in the endothelial cells of cerebral blood vessels warrants further investigation.

The water channel AQP4 can co-localize with Kir4.1 in glial cells^151^ and plays a significant role in potassium buffering.^152^ Converging evidence proposes that the absence of Dp71 hampers the clustering of AQP4 water channels in the glial endfeet near capillaries, leading to significant changes in water and ion balance in the brain, as well as alterations in vascular permeability and synaptic plasticity.^153,154^ Biochemical analysis revealed that the largest amount of AQP4 co-purified with Dp71 and β-dystroglycan, indicating that the membrane stability of AQP4 in this pool may depend on Dp71 or an integral DGC.^155^ A protocol that utilizes a wheat germ agglutinin (WGA)-coupled resin to purify the dystroglycan complex was used to analyse the possible interaction between AQP4 and the DGC.^156,157^ In brain lysates obtained from Wistar adult rats, AQP4 interacted with α-syntrophin, β-dystroglycan, Kir4.1, connexin-43, GFAP and vinculin. The results were confirmed by western blot analysis.^155^ AQP4 and Kir4.1 channels interact with Dp71 via syntrophins in glial cells in the brain and retina of *mdx3cv* mice.^158^

The altered distribution of AQP4 channels in cerebellum,^159^ hippocampus and cortex^160,161^ has been associated with cognitive and neurophysiological defects in animal studies.^71^ It must be noted that a specific deficiency in Dp71, as observed in the Dp71-null mouse model, results in a significant decrease in the polarized distribution of AQP4 channels in perivascular glial endfeet in cerebellum, hippocampus and cortex.^160,161^ This may induce phenotypes such as impaired hippocampal synaptic plasticity, resulting in spatial learning deficits.^154^ However, residual expression of AQP4 and β-dystroglycan is found in dystrophin-negative glial endfeet surrounding capillaries, indicating that compensatory mechanisms involving other dystrophin-gene products are not responsible for this expression. Interestingly, Dp427 was detected in hippocampal precapillary arterioles of Dp71-null mice.^162^ Other dystrophin or utrophin isoforms might contribute to the partial maintenance of AQP4 channels in glial endfeet associated with arterioles.

Fujimoto and colleagues^83^ assessed the physical and spatial associations between Dp71 and AQP4, as well as Kir4 channels, in both BG cells located in the PC layer and astrocytes. The co-precipitations of these channels were more efficient in cerebellum extracts compared with forebrain extracts, suggesting that interactions between Dp71 and these channels occur not only in perivascular astrocytes, which are distributed throughout the entire brain, but also in BG fibres specifically found in cerebellum and cortex. In line with this perspective, previous reports have proposed that the extracellular matrix protein, laminin, may enhance Kir4.1 clustering,^163^ and this effect might be mediated by the Dp71-dystroglycan molecular complex. Therefore, signals from extracellular matrices and surrounding cells might play crucial roles in establishing polarized expressions and proper functions of AQP4 and Kir4.1 channels. However, it is worth noting that AQP4 was observed at various positions, including the astrocytic glia limitans along the blood–brain barrier, BG processes and astrocytic processes within the cerebellar granule cell layer.

Numerous *in vitro* and *in vivo* studies have demonstrated the significance of the Kir4.1 channel as the primary mediator of glial potassium buffering.^164-171^ Therefore, Dp71 could potentially contribute to neuronal regulation through glia-mediated processes by interacting with AQP4 and Kir4.1 channels. Although the underlying molecular mechanisms require further elucidation, it is important to mention that Dp71-null mice exhibit selective enhancement of excitatory transmission at glutamatergic synapses formed by climbing fibres on PCs but not at those formed by parallel fibres.^159^

### Dysbindin and Major prion protein

Dp71 has been characterized to interact with dysbindin, although it has not been specified in which cell types the interaction takes place.^172^ Dysbindin, a coiled-coil-containing protein, binds to both α- and βDb in skeletal muscle and brain and interacts with the Dp71 isoform.^172^ Dysbindin is encoded by the *DTNBP1* (dystrobrevin binding protein) gene, which was first identified as a schizophrenia risk allele.^173^ Polymorphisms in *DTNBP1* have been associated with altered emotional working memory.^174^ Dysbindin has a role in intracellular protein trafficking involving organelles and lysosomes as well as synaptic homeostasis.

Benson and colleagues^172^ showed that Dp71 co-immunoprecipitates with dysbindin in adult rat brain. Immunoperoxidase-stained human brain sections were used to localize dysbindin,^175^ with immunoreactivity detected almost exclusively in axons; dysbindin distribution overlaps partly with the localization of βDb, notably in the brainstem.^120^ The concentration of dysbindin in axon terminals suggests a presynaptic localization. Ghiani and Del’Angellica^175^ also showed a complex of dysbindin, dystrobrevin and utrophin in the brain. However, the results obtained by Benson *et al*.^172^ have since been questioned by Nazarian and colleagues.^176^ At greater length, the later study did not indicate any specific association between dystrophin and dysbindin in rodent brain. Therefore, further experiments are needed to determine the association between dysbindin and dystrobrevin within dystrophin-containing complexes.

PrP is encoded by *PRNP*, interacts with Dp71, is expressed most predominantly in the nervous system, among many other tissues, and is involved in synaptic function and plasticity.^177,178^ Prions cause transmissible neurodegenerative diseases such as scrapie and bovine spongiform encephalopathy.^179^ PrP interacts with nNOS, α-tubulin, Dp71, α-syntrophin, synaptophysin and glutamic acid decarboxylase (GAD), although β-dystroglycan and β-actin have much stronger interactions as revealed by the immunoprecipitation of Syrian hamster brain samples.^180^ The interaction of dysbindin and PrP with Dp71 could potentially contribute to the organization and stabilization of synapses. Dysbindin's presynaptic localization coupled with PrP’s pre- and post-synaptic localization proposes a synergistic effect on synaptic maintenance and signalling. The interactions between these proteins may suggest that they are part of larger protein complexes, including Dp71, dystrobrevin and other associated proteins, likely involved in maintaining the structural integrity of synapses and facilitating efficient signal transmission. Given the reduction of dysbidin expression in schizophrenia^173^ and the role of PrP in transmissible neurodegenerative diseases,^179^ their interactions with Dp71 could have significant implications for understanding the molecular mechanisms underlying these conditions. Disruptions in these interactions might contribute to the pathogenesis of these disorders.

### Dp71–protein interactions: conclusions

Dp71 stands out as the most predominant dystrophin isoform in the brain, playing critical roles in both neuronal and glial functions. The complex interactions of Dp71 with proteins such as αDb, βDb, dystroglycan, AQP4 and other synaptic molecules underscore its significance in synaptic transmission and cellular processes. The differential localization of Dp71 in inhibitory post-synaptic compartments and its association with key proteins like Insyn1, Db and syntrophin highlights its specialized function in inhibitory synapses.

Future research should focus on elucidating the precise mechanisms by which Dp71 and its associated protein complexes regulate synaptic stability and function. Investigating the interplay between Dp71 and proteins involved in ion channel regulation, such as Kir4.1 and AQP4, could reveal new insights into glial-mediated neuronal regulation and potassium buffering. Additionally, the role of Dp71 in pathological conditions, including its interactions with Insyn1, dysbindin and major prion protein, warrants further exploration to understand its implications in neurodegenerative diseases and mental disorders that may be present in DMD patients.

Ultimately, advancing our understanding of Dp71's role in the brain through detailed molecular and functional studies will pave the way for targeted therapeutic strategies in conditions where dystrophin isoforms are implicated. Emphasizing the timing of expression, localization and interaction with other proteins will be crucial in deciphering the full spectrum of Dp71's role in maintaining neuronal and glial health.

### Dp40

Dp40 is the shortest dystrophin isoform, and its 5′-UTR originates from the same promoter as Dp71, located within intron 62 of the *DMD* gene, but the 3′-UTR is derived from the intron 70 sequence, and both present similar expression patterns, being the main products of *DMD* expression in the brain and retina.^13,69^ Dp40 partially localizes to the nucleus in PC12 cells^14,77^ and appears to be essential for PC12 neuronal differentiation,^181^ with a crucial role in neuronal maturation and presynaptic function^82^; it has been found to interact with SNAP receptor (SNARE) proteins,^81^ which play an important role in facilitating the fusion of biological membranes. Syntaxin-1a, SNAP25 and vesicle-associated membrane protein 2 (VAMP2) are SNARE proteins involved in regulating synaptic vesicle exocytosis in neurons and have been shown to interact with Dp40 in mouse brain, as identified using a specific in-house raised antibody against the N-terminal sequence with affinity chromatography and LC-tandem MS.^81^ Syntaxin1A is localized at the plasma membrane of presynaptic neuronal terminals and predominantly involved in vesicle fusion, while VAMP2 is anchored to the synaptic vesicle via a single transmembrane domain and SNAP-25 is a membrane-associated protein found in the active zone.^182-184^

Immunoprecipitation analysis revealed that syntaxin1A and SNAP25 interact similarly with Dp40 in brain lysate, implying Dp40's potential involvement in presynaptic terminal functions, such as exocytotic vesicle docking or fusion with the plasma membrane.^79^ Further immunohistochemistry analysis with an N-terminal specific antibody against Dp71 and Dp40 demonstrated co-localization of VAMP2 with short isoforms of dystrophin, including Dp40, in cultured mouse hippocampal neurons, strengthening the candidacy of VAMP2 as a protein that interacts with Dp40 in the presynaptic region.^79^ However, it is possible that a distinct Dp40 protein complex might also exist in the presynapses, separate from the SNARE complex.^79^

A study by Ohyama and colleagues^185^ reported that Calcium/calmodulin-stimulated protein kinase II (CaMKII), an abundant synaptic signalling protein essential for memory formation and the induction of synaptic potentiation, interacts with syntaxin1A and SNAP25 but not with VAMP2. Interestingly, using LC-MS/MS and immunoblot analysis, Tozawa *et al*.^81^ detected CaMKII as a molecule that interacts with Dp40, suggesting potential regulatory roles of Dp40 in Ca^2+^-triggered processes. At the same time, Dp40 may be localized to the nucleus and have a putative nuclear function, since prominent nuclear staining was found in primary hippocampal mouse neurons using an antibody against the N-terminal sequence. It is worth reporting that SNAREs not only participate in synaptic vesicle docking and fusion to the active zone but also play a critical role in the Ca^2+^-triggering step itself, adding another layer of significance to the interaction of Dp40 with these proteins.^186^

### Dp40–protein interactions: conclusions

Dp40, the shortest dystrophin isoform, is crucial to brain function, particularly in neurodevelopment and synaptic activity. Its localization to the nucleus in PC12 cells and interaction with SNARE proteins such as syntaxin-1a, SNAP25 and VAMP2 underscore its role in neuronal differentiation, maturation and synaptic vesicle exocytosis. Dp40's interaction with CaMKII highlights its involvement in synaptic potentiation and memory formation.

Future research should delve into Dp40's precise mechanisms of interaction with SNARE proteins and its potential nuclear functions. Understanding these interactions will be essential for developing therapeutic strategies for the brain comorbidities present in DMD patients.

### Dp140

Dp140, a dystrophin short isoform, is highly expressed in the CNS from the fetal period to infancy and is temporarily co-expressed with genes involved in early neurodevelopmental processes.^69^

Dp140/α1-syntrophin and the neuronal CaV2.1 channel molecular complex have been characterized, highlighting a potential role in neurotransmitter release at synapses.^187^ This unique molecular interaction involves the CaVβ4e subunit, which regulates CaV2.1 channel activity in the plasma membrane.^187^ Another crucial component in presynaptic membrane organization is the auxiliary subunit CaVα2δ-1. Immunoprecipitation analysis revealed that CaV2.1 interacts with dystrophin and CaVα2δ-1. Notably, the Dp140 isoform was the only dystrophin isoform to co-immunoprecipitate with CaVα2δ-1, despite Dp71's higher abundance in the brain. The release of neurotransmitters via CaV2.1 channels relies on the interaction between CaVα2δ-1 and α-neurexin,^188^ a known positive regulator of CaV2.1 channels that in turn interacts with α-dystroglycan.^189^ The opening of CaV channels due to increased Ca^2+^ triggers spontaneous glutamatergic release. This indicates that glutamatergic transmission is a process in which CaV2.1 and CaVα2δ-1 are involved. The proximity ligation assay results support this speculation since the interaction between Dp140 and the CaV2.1/CaVα2δ-1 complex has a somatodendritic localization in hippocampus and cerebellum.^187^ However, further studies are needed to uncover the molecular interactions of dystrophin with CaV channels within the nervous system. This understanding will provide deeper insights into how their dysregulation might contribute to neurological deficiencies observed in various human diseases, including DMD. Moreover, *in vitro* experiments in the same study utilizing HEK293 cells suggested that Dp140/α-syntrophin may exert a pivotal role in the membrane clustering of CaV2.1, presenting an intriguing implication.^187^

Recent studies have also shown that the absence of Dp140 impairs glutamatergic transmission in basolateral neurons of the amygdala in *mdx52* mice.^60^ In particular, it has been reported that there is a decrease in the excitatory/inhibitory post-synaptic current ratio and the frequency of miniature excitatory post-synaptic currents. These data show that synapse plasticity is different in mice lacking Dp140. Furthermore, the absence of Dp140 decreases the expression of Vesicular glutamate transporter 1 (VGLUT1), which is essential for glutamate release at presynaptic terminals, and, consequently, the number of presynaptic vesicles.^60^

The correlation between the observed impairment in glutamate release and the lack of Dp140 in knockout mice can be understood through several interconnected mechanisms. In more detail, Dp140 is expressed not only in neurons but also in astrocytes, which play a critical role in regulating synaptic transmission, including glutamate release. Astrocytes are involved in the uptake, recycling and release of glutamate, and the loss of Dp140 in these cells could disrupt these processes, leading to impaired glutamate signalling.

This highlights a potential astrocyte-mediated mechanism by which Dp140 influences presynaptic glutamate release. The impairment in glutamate release due to the lack of Dp140 can also lead to broader dysfunction in neural networks, particularly in brain regions like the BLA, which is involved in emotional and cognitive processing. This could contribute to the neurodevelopmental abnormalities observed in DMD patients. These studies shed light on the intricate molecular interactions between syntrophins, dystrophin family proteins and the CaV2.1 channel complex, providing valuable insights into their roles in neurological disorders and synaptic function in the brain. In conclusion, Dp140, through its interaction with α1-syntrophin, helps to organize and stabilize the CaV2.1 channel complex in presynaptic terminals. This organization ensures efficient calcium influx, which is essential for the proper release of glutamate. The presence of the CaVβ4e subunit further enhances this process, indicating that Dp140 is crucial for fine-tuning synaptic transmission, particularly in neurons where glutamatergic signalling plays a vital role. The absence of Dp140 disrupts this complex, leading to impaired glutamate release and synaptic dysfunction.

Dp140 interacts also with the proteins described earlier as Dp71 interactors. Specifically, β-dystroglycan immunoprecipates are enriched in Dp140^106^ and βDb co-immunoprecipitates with Dp140 in brain lysates.^119^ The functional implications of these interactions in the CNS remain ambiguous, and further investigation is needed.

### Dp140–protein interactions: conclusions

Dp140 may significantly influence early neurodevelopmental processes, although its expression in early embryonic stages has not been fully characterized to date. Its high expression and temporary co-expression with early neurodevelopmental genes underscores its importance in the maturation and function of the brain. The interaction of Dp140 with the CaV2.1 channel complex and α1-syntrophin at the plasma membrane highlights its importance in selective neurotransmitter release, particularly affecting glutamatergic transmission in BLA neurons. This interaction is crucial for synaptic plasticity, as evidenced by the regulation of CaV2.1 channel activity via the CaVβ4e subunit and the integral role of the auxiliary subunit CaVα2δ-1 in presynaptic membrane organization.

Notably, the co-immunoprecipitation of Dp140 with CaVα2δ-1, despite the higher abundance of Dp71, highlights the unique and specific role of Dp140 in synaptic organization and function. The somatodendritic localization of the Dp140/CaV2.1/CaVα2δ-1 complex in hippocampus and cerebellum further supports its involvement in glutamatergic transmission. Recent findings indicate that the absence of Dp140 disrupts glutamatergic transmission in the BSA of *mdx52* mice, as evidenced by altered excitatory/inhibitory post-synaptic current ratios and reduced frequencies of miniature excitatory post-synaptic currents. This disruption also correlates with decreased levels of VGLUT1 and a reduction in presynaptic vesicles, highlighting the impact of Dp140 deficiency on synaptic plasticity. The absence of Dp140 (and Dp427) in *mdx52* mice, leading to impaired glutamatergic transmission and altered synaptic plasticity, emphasizes its critical function in maintaining excitatory/inhibitory balance and presynaptic vesicle number.

The precise molecular mechanisms governing Dp140's interactions with the CaV2.1 channel complex and other proteins such as β-dystroglycan and syntrophins need to be investigated. Understanding how Dp140 influences synaptic plasticity and neurotransmitter release could illuminate potential therapeutic targets for neurological disorders. Exploring the impact of Dp140 on the expression of synaptic proteins like VGLUT1 and the regulation of presynaptic vesicles will be crucial to advancing our knowledge of synaptic function and plasticity in the CNS.

## Implications of various dystrophin isoform deficiencies on CNS features and comorbidities in DMD

As described earlier, dystrophin isoforms are expressed in several cell types; therefore, mutations in different regions could lead to variable phenotypes. Various combinations of intellectual disability and neurobehavioural comorbidities occur in one-third of boys with DMD, and multiple studies have highlighted the roles of dystrophin isoforms in these CNS features.^45,190^ In more detail, in a cohort of 62 DMD patients, 15% of boys lacking only Dp427 had intellectual disability, compared with 25% of boys lacking Dp427 and Dp140 and 64% of boys lacking Dp427, Dp140 and Dp71.^191^ Evidence gathered from a combination of studies indicates a role for dystrophin and the DGC at inhibitory post-synapses, along with the evident developmental role of Dp140, which may explain some of the cognitive comorbidities associated with DMD. On the contrary, neuropsychiatric aspects such as ASD and ADHD can be found across the spectrum of mutations, pointing towards a role for Dp427.^44,45,192^ Enhanced anxiety and fear response are also reported in children with DMD, regardless of their genotype, further supporting a role for Dp427 in emotional disturbances.^45,193^

A variety of animal studies have also identified the CNS comorbidities related to DMD; *mdx52* mice display higher anxiety and more impaired fear response, learning and memory than *mdx23* mice, indicating that Dp140 loss increases emotional disturbances.^194^ Another study that investigated ASD-like behaviours in *mdx52* mice lacking Dp140 showed reduced glutamatergic transmission in the BLA pyramidal neurons compared with wild-type and *mdx* mice. This implies a role for Dp140 in ASD-related behaviours.^60^ Interestingly, dystrophic mice lacking both Dp427 and Dp140 exhibit more severe motor dysfunction, indicating that Dp140 may contribute to coordination and motor performance; a similar finding applies to boys with DMD.^190^

RNA therapies to restore Dp427 or Dp140 have been associated with phenotypic improvement in the relevant mouse model, opening a new avenue for potential therapeutic interventions. Specifically, antisense oligonucleotide made of phosphorodiamidate morpholino oligomer (PMO) led to a partial restoration of brain Dp427 and an improvement in the characteristic fear-motivated defensive behaviour of *mdx23* mice.^62^ Recent studies showed that administering tricycloDNA-antisense oligonucleotide targeting *DMD* exon 51 via intracerebroventricular injection in the brain of *mdx52* mice resulted in partial Dp427 restoration in the brains of these mice, reducing anxiety and unconditioned fear.^195^ Additionally, injection of antisense oligonucleotides targeting *DMD* exon 53 or Dp140 mRNA-loaded polyplex nanomicelles into the BLA restored Dp140 expression and reduced abnormal social behaviour in *mdx52* mice.^60^

## Conclusions and future research

Dystrophin expression is well characterized in muscle; however, more work is needed to provide a fuller picture of the expression and function of the multiple dystrophin isoforms in the brain. Our review summarizes the proteins that interact with dystrophin in the brain. This review stresses that dystrophin's role extends beyond the musculature into crucial brain functions spanning neuronal development, AQP4 involvement in blood–brain barrier development, synaptic transmission and neuronal maturation. Patients with DMD commonly have associated brain function comorbidities such as ADHD, ASD, epilepsy and intellectual disability, which can be broadly related to the lack of individual isoforms. Understanding the functional role of dystrophin and its protein interactors in the brain is crucial, as it will provide mechanistic insight into the pathogenesis of DMD CNS comorbidities and novel avenues for developing potential therapeutic strategies to address the root cause of DMD. Indeed, multiple groups are now focusing on RNA therapies in preclinical models, and this review could be the prelude for future therapeutic interventions for brain involvement in DMD.

## Search strategy

We searched PubMed for articles published in English from 1 January 1994 to 1 January 2024, using the search terms ‘dystrophin’, ‘Duchenne’, ‘dystrophinopathy’, ‘interactors’, ‘mdx’ or the causative gene, ‘DMD’, and ‘brain’. This retrieved 215 articles published in the last 30 years. We generated the final reference list based on topics that fit the scope of this review and included landmark papers.
